# Introduction to This Special Issue of *Toxins*: Application of Novel Methods for Mycotoxin Analysis

**DOI:** 10.3390/toxins14030190

**Published:** 2022-03-04

**Authors:** Veronica M. T. Lattanzio, Biancamaria Ciasca

**Affiliations:** Institute of Sciences of Food Production, National Research Council of Italy, Via Amendola, 122/O, 70126 Bari, Italy; biancamaria.ciasca@ispa.cnr.it

Crop contamination by mycotoxins is a global problem that poses significant economic burdens due to the food/feed losses that are caused by reduced production rates; the resulting adverse effects on human and animal health and productivity; and the trade losses associated with the costs incurred by inspection, sampling, and analysis before and after shipments. In this scenario, the development of fit-for-purpose analytical methods for regulated and (re)-emerging mycotoxins continues to be a dynamic research area. Some of the current trends in this research area are presented in the papers that have been selected for this Special Issue of *Toxins*.

The collected contributions address either the need for improved methods for mycotoxin detection addressed by new or incoming regulation (ergot alkaloids and *Alternaria* toxins) as well as methods for the detection of multiple mycotoxins. New approaches to enhance the performance of well-established methodologies, such as the enzyme-linked immunosorbent assay (ELISA) and fluorescence polarization immunoassays (FPIA), have already been proposed.

The recently issued European Commission Regulation (EU) 2021/1399, which sets the maximum limits for the sum of 12 main ergot alkalois (EAs), has been a driver for the development of improved analytical approaches for monitoring and official control purposes. Analytical challenges related to EA detection have been discussed by Lattanzio et al. [1], who reported on EA monitoring data in cereal and cereal-derived products collected in Italy over the period of 2017–2020 for official control purposes. To this scope, the authors set up and applied a method upon the verification of its fitness for in-house validation purposes. Poapolathep et al. [2] explored the liquid chromatography tandem mass spectrometry (LC-MS/MS) performance and applicability of EA detection in swine and dairy feeds, revealing a significant number of contaminated samples, regardless of whether the contamination was at EU regulation-compliant levels. Indeed, this new regulation also calls for methods that can be implemented for quick compliance testing. An interesting approach for EA screening was proposed by Kuner et al. [3], whose proposed method is based on EA cleavage by hydrazinolysis to convert them in a lysergic acid derivative, allowing their total content (sum of 12 EA) in food and feed to be quantified. 

Though not yet regulated, *Alternaria* toxins (ATs) have been included in European Commission (EC) monitoring programmes since 2012. To fulfil this requirement and to complement the available LC-MS-based methods, research made steps toward the development of AT antibodies. Addante-Moya et al. [4] prepared and characterized two rationally designed synthetic haptens of *Alternaria* mycotoxins, which led to high-affinity antibodies of alternariol and alternariol monomethyl ether. These findings will pave the way for new immunoassay developments.

FPIA is a widely used homogeneous-based immunoassay with simple and rapid operational procedures. With the goal of keeping the sample preparation procedure as simple as possible, achieving improved sensitivity and selectivity is all about choosing the best tracer/antibody combination. In the study by Huang et al. [5], different antibody/tracer combinations were tested to set up a FPIA for the detection of ochratoxin A in rice. The resulting immunoassay fulfilled the mycotoxin screening and testing method validation and performance criteria set by the EU.

Evaluating contaminated food directly for specific fungi via the genes involved in aflatoxin production is a promising strategy. Based on this alternative approach, Elsayed Hafez et al. [6] developed a recombinant AflR gene (involved in aflatoxin biosyntesis) antiserum ELISA for the detection of aflatoxin-producing fungi in contaminated food.

Standardization is a challenging journey, especially when validating multi-mycotoxin methods. De Girolamo et al. [7] reported on this process within the M/520 standardization mandate of the European Commission. An LC-MS/MS method for the simultaneous determination of trichothecenes and zearalenone in wheat, wheat flour, and wheat crackers was validated through a collaborative study involving 15 participants from 10 countries. The results proved that the candidate method was fit for enforcement purposes. 

Overall, even though not comprehensive, the collected manuscripts provide an up-to-date picture of the current trends in novel mycotoxin analysis methods, with LC-MS continuing to be the technique of choice for multi-mycotoxin detection, and testing new immunoreagents (labels and/or antibodies) represents a key step for improving the performance of screening methods.
